# Severity of post-Roux-en-Y gastric bypass dumping syndrome and weight loss outcomes: is there any correlation?

**DOI:** 10.1007/s00423-022-02736-w

**Published:** 2023-01-06

**Authors:** Mohammad Kermansaravi, Shahab ShahabiShahmiri, Ramon Vilallonga, Barmak Gholizadeh, Amir Hossein DavarpanahJazi, Yeganeh Farsi, Rohollah Valizadeh, Masoud Rezvani

**Affiliations:** 1 Department of Surgery, Minimally Invasive Surgery Research Center, Division of Minimally Invasive and Bariatric Surgery, School of Medicine, Rasool-E Akram Hospital, Iran University of Medical Sciences, Tehran, Iran; 2 Iran National Center of Excellence for Minimally Invasive Surgery Education, Iran University of Medical Sciences, Tehran, Iran; 3 Obesity and Metabolic Surgery Unit, Vall Hebron Campus Hospital, Barcelona, Spain; 4 Department of General Surgery, Shahid Modarres Hospital, Shahid Beheshti University of Medical Sciences, Tehran, Iran; 5 Urmia University of Medical Sciences, Urmia, Iran; 6 Department of Surgery, Davis Hwy, Inova Fair Oaks Hospital, 14904 JeffersonSuite 205, Woodbridge, VA USA

**Keywords:** RYGB, Dumping syndrome, Weight loss, Gastric bypass, Bariatric surgery

## Abstract

**Purpose:**

The present research was conducted to evaluate the effect of the severity of dumping syndrome (DS) on weight loss outcomes after Roux-en-Y gastric bypass (RYGB) in patients with class III obesity.

**Methods:**

The present retrospective cohort study used the dumping symptom rating scale (DSRS) to evaluate the severity of DS and its correlation with weight loss outcomes in 207 patients 1 year after their RYGB. The patients were assigned to group A with mild-to-moderate DS or group B with severe DS.

**Results:**

The mean age of the patients was 42.18 ± 10.46 years and their mean preoperative BMI 42.74 ± 5.59 kg/m^2^. The total weight loss percentage (%TWL) in group B was insignificantly higher than that in group A, but besides that was not significantly different in the two groups.

**Conclusion:**

The present findings suggested insignificant relationships between the presence and severity of DS after RYGB and adequate postoperative weight loss.

**Supplementary Information:**

The online version contains supplementary material available at 10.1007/s00423-022-02736-w.

## Purpose

Roux-en-Y gastric bypass (RYGB) is currently one of the most frequent bariatric surgeries performed worldwide [1]. Dumping syndrome (DS) potentially occurring within the first 3 months of RYGB is characterized by unpleasant gastrointestinal symptoms, including abdominal pain, nausea and diarrhea, and vasomotor symptoms such as palpitation, hypotension, sweating, dizziness, fatigue, and flushing [2, 3]. DS is categorized as early and late with different etiologies. Early DS mostly occurring 10–30 min after meals through rapid emptying of hyperosmolar ingestion into the jejunum is caused by the absence of the pylorus in the alimentary tract [4]. Late DS occurring 1–3 h after meals due to high-insulin response after ingestion of high carbohydrate meals emerges as postprandial hypoglycemia [5].

Severe DS can theoretically cause the patient to avoid high-fat or high-carbohydrate foods, which is in line with dieticians’ nutritional orders, helps with weight loss and prevents weight regain after RYGB [6–8]. Given the rarely conducted studies on the effects of DS on weight loss outcomes after bariatric procedures [7, 8], the present study aimed at evaluating correlations between DS severity and weight loss outcomes 1 year after RYGB.

## Methods

### Design and setting

This retrospective cohort study recruited 207 patients undergoing primary RYGB at Rasool-e-Akram Hospital, Tehran, Iran, from January 2019 to August 2020. The inclusion criteria comprised a minimum age of 18 years and BMI > 40 kg/m^2^ or BMI > 35 kg/M^2^ and obesity-related comorbidities. The data extracted from the Iran National Obesity Surgery Database (INOSD) [9] included comorbidities and demographic and anthropometric information. Symptoms of DS were recorded through phone interviews with eligible candidates using the early dumping symptom rating scale (DSRS) within 1 year after the operation [10]. The exclusion criteria consisted of revisional and conversional RYGB and history of upper gastrointestinal surgeries. Out of the 11 items in the DSRS, nine relate to the symptoms emerging within 10–30 min after meals, one relates to the symptoms emerging after drinking during meals, and one to the symptoms after very sweet drinks. The severity of each symptom in the previous week was rated on a 7-point scale ranging from 1 “no trouble at all” to 7 “very severe problems.” The frequency of dumping symptoms within the previous 2 weeks was also measured on a 6-point scale ranging from 1 “no trouble at all” to 6 “several times a day.” In terms of severity, the patients with “no trouble at all,” “minor inconvenience,” “mild trouble,” or “moderate trouble” were assigned to group A and those with “quite severe problems,” “severe problems,” or “very severe problems” to group B. In terms of frequency, the patients with “no trouble at all,” “less than once a week,” “once a week,” or “a few times a week” were allocated to group A (less than once a day) and those with “once a day” or “several times a day” to group B (at least once a day).

### Surgical technique

An experienced surgical team performed long laparoscopic biliopancreatic RYGB in the French position. After creating a 6-cm slim gastric pouch with about 40–60 ml volume, a posterior gastro-jejunostomy was created with a 30-mm linear endo-stapler about 125 cm after the ligament of Treitz (long BP). A 45-mm jejuno-jejunal anastomosis was then created using the linear endo-stapler and a 75-cm Roux limb. Both Petersen and jejuno-jejunal defects were closed with non-absorbable sutures at the end of the operation.

Enhanced recovery after surgery (ERAS) protocol was applied to all the included patients and their majority was discharged within the first day after the surgery. All the patients were followed-up 10 days and 1, 3, 6, and 12 months after the surgery by the surgical, nutritionist, and multi-disciplinary team members.

### Statistical analysis

The data were analyzed in SPSS-21 at a *P* < 0.05 significance level. After confirming the distribution normality of the variables using the Kolmogorov–Smirnov test, the relationships of the main factors with the outcomes were investigated using the independent *t* test and linear regression. The data were expressed as descriptive statistics, i.e., frequency and percentage, or mean ± SD.

## Results

The mean age of the patients was 42.18 ± 10.46 years, their mean preoperative BMI 42.74 ± 5.59 kg/m^2^ and 89.4% (*n* = 185) were female. Table 1 presents baseline variables, i.e., diabetes mellitus (T2DM), obstructive sleep apnea (OSA), cardiovascular disease (CVD), gastroesophageal reflux disease (GERD), hypertension (HTN), and dyslipidemia (DLP).

**Table 1:** Demographic and clinical characteristics of the *studied* patients

Variable	Value
Age, mean ± SD (range), year	42.18 ± 10.46 (21–72)
Female sex, no. (%)	185 (89.4)
Pre-op BMI, mean ± SD (range), kg/m^2^	42.74 ± 5.59 (31.08–55.68)
T2DM, no. (%)	38 (18.4)
OSA, no. (%)	81 (39.1)
CVD, no. (%)	4 (1.9)
HTN, no. (%)	47 (22.7)
DLP, no. (%)	61 (29.5)
GERD, no. (%)	55 (26.57)

*BMI* body mass index, *SD* standard deviation, *T2DM* type 2 diabetes mellitus, *OSA* obstructive sleep apnea, *CVD* cardiovascular disease, *GERD* gastroesophageal reflux disease, *HTN* hypertension, *DLP* dyslipidemia.

Table 2 shows the difference for TWL% in the groups A and B regarding the severity of the measured items. These TWL% were not significantly different (*P* > 0.05) but fatigue and cramp.

**Table 2:** Comparison of TWL% by the severity of the dumping syndrome

Item		%TWL	*P* value
		Mean ± SD	
Fatigue 10–30 min after meals	Group A	33.06 ± 1.20	0.017
	Group B	32.52 ± 1.49	
Palpitations 10–30 min after meals	Group A	32.90 ± 1.30	0.276
	Group B	33.13 ± 1.22	
Sweating/flushing 10–30 min after meals	Group A	32.91 ± 1.26	0.367
	Group B	33.11 ± 1.37	
Cold sweats/paleness	Group A	33.00 ± 1.20	0.145
	Group B	32.62 ± 1.71	
Need to lie down for a while	Group A	32.97 ± 1.21	0.694
	Group B	32.89 ± 1.46	
Diarrhea 10–30 min after meals	Group A	32.96 ± 1.30	0.831
	Group B	32.90 ± 1.20	
Nausea/vomiting 10–30 min after meals	Group A	38.94 ± 1.22	0.777
	Group B	33.00 ± 1.53	
Stomach cramps 10–30 min after meals	Group A	33.05 ± 1.21	0.023
	Group B	32.53 ± 1.52	
Fainting esteem/shaking 10–30 min after meals	Group A	32.99 ± 1.22	0.438
	Group B	32.78 ± 1.63	
Pain/vomiting after drinking fluids in moderate amount	Group A	33.03 ± 1.26	0.320
	Group B	32.86 ± 1.30	
Sudden abdominal problems, faint, or fatigue after drinking heavily sweetened fluids	Group A	32.96 ± 1.22	0.921
	Group B	32.94 ± 1.37	

*Group A: mild to moderate, **Group B: severe

Table 3 shows differences in %TWL between groups A and B by the frequency of the measured items. Although %TWL was insignificantly different (*P* > 0.05), nausea or vomiting and cold sweats were significantly different between the two groups.

**Table 3:** Comparing %TWL by the DS frequency

Item		%TWL	*P* value
		Mean ± SD	
Fatigue	Group A	33.00 ± 1.19	0.052
	Group B	32.39 ± 1.97	
Palpitations	Group A	32.98 ± 1.29	0.104
	Group B	32.27 ± 0.81	
Sweating/flushing	Group A	32.97 ± 1.29	0.331
	Group B	32.54 ± 0.85	
Cold sweats/paleness	Group A	33.00 ± 1.20	0.001
	Group B	31.26 ± 2.43	
Need to lie down	Group A	32.98 ± 1.18	0.357
	Group B	32.73 ± 1.94	
Diarrhea	Group A	32.95 ± 1.28	0.679
	Group B	33.17 ± 1.59	
Nausea/vomiting	Group A	33.00 ± 1.20	0.009
	Group B	31.80 ± 2.45	
Stomach cramps	Group A	32.96 ± 1.30	0.603
	Group B	32.72 ± 0.79	
Fainting/shaking	Group A	32.96 ± 1.12	0.854
	Group B	32.91 ± 1.68	

Tables 4 and 5 show insignificant differences in %TWL between the two groups by the severity and frequency of DS (*P* > 0.05), it should be noted that both Tables 4 and 5 included 207 patients (187 + 20 patients = 207 in Table 4 and 193 + 14 = 207 patients in Table 5), but the difference in the “mild-to-moderate” and “severe” groups is related to the assessment of the DS in terms of “severity” or “frequency,” e.g., severe score in Table 4 is more than 44 but in Table 5 is more than 27 indeed type of scoring is different so it is rational to have different patients in each group. Eleven severity items scored 1–7 (total score = 11–77) were considered mild-to-moderate (11–44) or severe (more than 44). Nine frequency items scored 1–6 (total score = 9–54) were also considered mild-to-moderate (9–27) or severe (more than 27).

**Table 4:** Comparing %TWL by the severity of DS

Severity		*N*	Mean ± SD	*P* value
%TWL	Mild-moderate (11–44)	187	32.95 ± 1.29	0.959
	Severe (more than 44)	20	32.96 ± 1.16

**Table 5:** Comparing %TWL by the frequency of DS

Frequency		*N*	Mean ± SD	*P* value
%TWL	Mild-to-moderate (9–27)	193	32.95 ± 1.29	0.929
	Severe (more than 27)	14	32.98 ± 1.15	

According to Table 6, gender, age, DS, and comorbidities such as T2DM, DLP, HTN, and OSA at 1-year follow-up were not correlated with %TWL. Intractable DS was not observed in any of the patients, which required reversal to normal anatomy or sleeve, and all the patients were treated with conservative managements including diet modifications and medical treatment.

**Table 6:** Predictive factors for %TWL obtained using linear regression

Variable		Non-standardized coefficient		Standardized coefficient	*t*	*P* value
		*B*	Std. error	Beta		
Age	To predict %TWL	0.012	0.014	0.110	0.832	0.409
Gender	To predict %TWL	− 0.843	0.424	− 0.248	− 1.988	0.052
T2DM at 1-year follow-up	To predict %TWL	0.158	0.484	0.044	0.327	0.745
Dyslipidemia at 1-year follow-up	To predict %TWL	− 0.606	0.358	− 0.227	− 1.693	0.096
Hypertension at 1-year follow-up	To predict %TWL	0.022	0.468	0.007	0.047	0.962
OSA at 1-year follow-up	To predict %TWL	0.951	0.848	0.140	1.121	0.267

## Discussion

Dumping syndrome is a well-known sequel of Roux-en-Y gastric bypass (RYGB). Studies have reported a prevalence of DS ranging from 3 to 80% after LRYGB [4, 5, 11–13]. DS can also be seen after non-anastomotic bariatric procedures, such as sleeve gastrectomy (SG). DS after SG may occur in up to 40% of patients [4, 5, 11–13].

In summary, our study showed that the mean age and pre-op BMI of the patients were 42.18 ± 10.46 years and 42.74 ± 5.59 kg/m^2^, respectively. Study groups of “mild to moderate” and “severe” were not significantly different (*P* > 0.05) regarding %TWL but fatigue and cramp. Regarding frequency of “less than 1 time per day” and “once and more than 1 time per day,” %TWL was not significantly different (*P* > 0.05) but nausea vomiting feelings and cold sweats. It should be noted that severity with 11 questions ranging from 1 to 7 (minimum total score, 11; maximum total score, 77) was divided into mild to moderate (score, 11–44) and severe (more than 44). The frequency with 9 questions ranging from 1 to 6 (minimum total score, 9; maximum total score, 54) was divided into mild to moderate (score, 9–27) and severe (more than 27). None of the comorbidities such as T2DM, dyslipidemia, HTN, and sleep apnea at 1-year follow-up, gender, and age as well as dumping syndromes were correlated with the changes of %TWL. None of the patients included had intractable DS which could require reversal to normal anatomy or sleeve gastrectomy.

According to the DSRS self-assessment questionnaire, we measured the severity and frequency of nine dumping symptoms. In our study, as explained, subjects were classified based on the results of the DSRS questionnaire into two groups: mild to moderate and severe dumping syndrome. The incidence of severe DS in our study population following LRYGB was 20 cases (10%) while the other 187 cases (90%) complained of mild to moderate dumping syndrome. Similarly, in Laurenius et al.’s study among 129 patients post-LRYGB, 12% reported severe dumping problems based on the DSRS questionnaire [10].

The DS was thought to induce changes in food preference, as initially, it was considered as a useful characteristic of the RYGB to “teach” patients to avoid calorie-dense foods and thus consume fewer calories. However, an unlikely explanation as most patients with severe dumping still reports that they have learned to consume only small quantities that do not cause negative visceral symptoms or consume sweets at night before bedtime.

The present study did not find relationships between DS and weight loss. In addition to the insignificantly-higher %TWL in group B (severe DS) than in group A (mild-to-moderate), the difference between the two groups was insignificant in terms of %TWL at one-year follow-up by the severity and frequency of each measured dumping symptom. Given the lack of differences in the eating habit of the two groups, the patients might have resumed their preoperative diet.

Several published studies could not show any significant association between dumping and concomitant weight loss. A study by Banerjee et al. recruiting 50 patients undergoing RYGB [13] found higher decreases in the mean BMI in the patients without DS compared to in those with DS 1 year (18.5 versus 14.4) and 2 years (17.8 versus 13.7) after the RYGB. In contrast, in Looveren et al. study on 100 consecutive patients who underwent revisional RYGB, due to inadequate weight loss or band intolerance after laparoscopic adjustable gastric banding (LAGB), fifty-five patients (59.1%) suffered from dumping. Based on the findings in their patient group, we suggest that dumping helps patients achieve sustainable weight loss. Therefore, dumping can be regarded as a positive sequel rather than a complication [8].

A study by Laurenius et al. on early dumping found patients to consider DS a shield against over-consumption despite their unpleasant symptoms [14–17]. Few studies on late DS also reported associations between the symptoms of post-bariatric hypoglycemia and weight regain [7, 15, 18].

Different degrees of DS were observed in all the present study patients after RYGB. Mild-to-moderate DS might have been, however, under-reported in the absence of specific screening tools.

The present study limitations comprised failure to perform objective tests to confirm DS given the limitation of using questionnaires. This study relied on patients’ phone-interview self-reports in the DSRS to diagnose DS. In addition, DSRS does not differentiate between early and late dumping syndromes, but is rather screening for early dumping symptoms. As the best predictor of overall health, long-term weight loss and weight maintenance, dietary habits were not investigated. It is well known that the dietary habits of an individual are the best predictors of their overall health and long-term weight loss and weight maintenance. DS after gastric bypass was found not to guarantee adequate weight loss.

In conclusion, the risk of DS causes significant apprehension in patients, even to the point of declining surgery preoperatively in some cases. DS and its severity were found not to help with weight loss. Dietary habit modification appeared the optimal predictor of weight loss in the long-run.

### Supplementary Information

Below is the link to the electronic supplementary material.

Supplementary file1 (DOCX 41 KB)

## Data Availability

The datasets generated and/or analyzed in the present study are made available by the corresponding author upon a reasonable request.
